# Practical Notes on Diagnosis and Treatment

**Published:** 1910-10-22

**Authors:** 


### Practical Notes on Diagnosis and Treatment.

**Granular Kidney.**

In granular kidney it is better that the pulse tension should be high than low. Low tension only occurs in the later stage of the disease, though it may continue for some time.—*Dr. Henry Green.*

**Rheumatoid Arthritis and Gout.**

I lay stress on the fact of exostoses in true gout, and on that which is even more noteworthy—the occurrence of true ankylosis or bony synostosis in this disease—a condition which we do not find in rheumatoid arthritis.—*Sir Dyce Duckworth.*

**Rectal Examination of the Prostate.**

Although a time-honoured custom, rectal examination in cases of prostatic enlargement has been accorded an exaggerated value. The size of the prostate is no gauge of its obstructive power. What you have to do is to determine how far the urinary outflow is obstructed, and this can only be discovered by the passage of a catheter. Mere rectal palpation cannot inform you whether the urinary outlet is interfered with.—*Mr. W. Bruce Clarke.*

**Malignant Disease of the Liver.**

A large cirrhotic liver is likely to be confused with malignant disease in a comparatively early stage, but only at this period, for cirrhosis never produces the extreme enlargement seen in advanced cases of malignant disease. Enlargement extending below the iliac crest is, provided a movable or displaced liver is eliminated, almost certainly malignant.—*Dr. H. D. Rolleston.*

**Uterine Prolapse.**

The sequence of events in any case of prolapse is usually this: the first organ to descend is the bladder, pushing the anterior vaginal wall before it and so causing a cystocele. This puts traction on the cervix and retroverts the uterus. Traction continuing, the uterus descends and drags with it the posterior vaginal wall. This strips the posterior vaginal wall off the rectum, the peritoneum descending to take a lower attachment to the rectum and so the prolapse becomes complete. In a small percentage of cases, however, a rectocele precedes the uterine descent, or a rectocele and cystocele occur together.—*Dr. T. G. Stevens.*

